# PD147 Overview Of Multiplex Antigen Near-Patient Tests For Acute Respiratory Infections

**DOI:** 10.1017/S0266462324003817

**Published:** 2025-01-07

**Authors:** Tom Lynch, Cillian McDowell, Eimear Burke, Carol McLoughlin, Michelle O’Neill, Susan Spillane, Máirín Ryan

## Abstract

**Introduction:**

Multiplex antigen near-patient tests (NPTs) can detect multiple virus-specific antigens during acute infection. This project aimed to provide an overview of evidence on the effectiveness, advantages and disadvantages, and feasibility of multiplex antigen NPTs to identify common respiratory pathogens (including SARS-CoV-2 and one or both of the influenza and respiratory syncytial viruses) in residential and primary care settings.

**Methods:**

A non-systematic literature search was conducted on the 28 July 2023 in the MEDLINE, Embase, Cochrane Library, and ClinicalTrials.gov databases to identify relevant literature. National and international agency websites were searched for guidance or recommendations relating to multiplex antigen NPTs. Relevant citations were screened and extracted by one reviewer and cross-checked by a second reviewer. Health technology assessments, systematic reviews, observational studies, and randomized and non-randomized controlled trials were considered eligible for inclusion in the overview. No formal quality appraisal of included documents was conducted. Due to the variation in study types, study findings were narratively assessed.

**Results:**

Ten documents were identified in total. One complete prospective evaluation and seven incomplete clinical trials were identified. No relevant primary studies were identified for effectiveness outcomes such as time to appropriate treatment with antibiotics or antivirals. An evaluation published by the Haute Autorité de Santé in France reported that there was insufficient performance data regarding multiplex antigen NPTs in clinical practice. In a joint statement on respiratory virus testing, the Public Health Laboratory Network and Communicable Diseases Network in Australia indicated that antigen NPTs were not recommended due to their poor ability to identify influenza A.

**Conclusions:**

Evidence on the effectiveness, advantages, disadvantages, and feasibility of multiplex antigen NPTs to detect common respiratory pathogens in primary and residential care settings is sparse. Studies are required to assess the diagnostic performance (relative to reverse transcription polymerase chain reaction tests) and clinical utility of multiplex antigen NPTs in residential and primary care settings.

